# Case Report: Pathologic complete response in a patient with simultaneous diagnoses of resectable NSCLC and myeloid neoplasm with PDGFRA rearrangement treated with concurrent neoadjuvant chemoimmunotherapy and imatinib: translating clinical trial data to real-world practice

**DOI:** 10.3389/fonc.2025.1589126

**Published:** 2025-07-28

**Authors:** Chad B. Sussman, Minal Shah, Kausha Amin, Rachel Fanaroff, Eric Krause, Vu H. Duong, Samuel Rosner

**Affiliations:** ^1^ Department of Medicine, University of Maryland School of Medicine, Baltimore, MD, United States; ^2^ Medical Oncology and Hematology, Medstar Health, California, MD, United States; ^3^ Department of Pathology, University of Maryland (UM) Capital Region Health, North Largo, MD, United States; ^4^ Department of Pathology, University of Maryland School of Medicine, Baltimore, MD, United States; ^5^ Department of Surgery, University of Maryland School of Medicine, Baltimore, MD, United States; ^6^ Department of Medicine, University of Maryland Marlene and Stewart Greenebaum Comprehensive Cancer Center, Baltimore, MD, United States

**Keywords:** NSCLC, PDGFRA, neoadjuvant immunotherapy, nivolumab, imatinib

## Abstract

Neoadjuvant chemoimmunotherapy has become an established treatment approach in resectable non-small cell lung cancer (NSCLC). The utilization of neoadjuvant immune checkpoint blockade (ICB) and coordination of care in the real-world setting present important challenges, with limited data available for patients with multiple synchronous primary malignancies. This case describes a 57-year-old man with simultaneous diagnoses of resectable stage IIIA NSCLC and *PDGFRA*-rearranged myeloid neoplasm who received neoadjuvant chemotherapy and nivolumab in combination with imatinib prior to definitive resection. The treatment course was uncomplicated, resulting in a complete pathologic response and resolution of the eosinophilia. Our report highlights the decision-making involved in pursuing combined systemic therapy of the patient’s multiple malignancies and in navigating barriers related to tissue availability for biomarker testing. Approaches to neoadjuvant immunotherapy in early-stage NSCLC can be successful but must remain adaptable to reliably manage complex patient presentations in real-world, non-clinical trial settings.

## Background

Recent advances in the management of early-stage non-small cell lung cancer (NSCLC) have led to FDA approval of several neoadjuvant and perioperative regimens incorporating platinum-doublet chemotherapy with immune checkpoint blockade (ICB) prior to surgical resection in patients without sensitizing *EGFR* or *ALK* alterations (1–3). Outcomes from pivotal phase III studies have demonstrated significant improvements in pathologic response (1–4), event-free survival, and now overall survival (3) with the incorporation of ICB in patients with resectable disease. With the increasing uptake of this neoadjuvant ICB paradigm, reports detailing real-world experiences of complex treatment planning for patients with competing comorbidities are needed to guide clinicians in this rapidly evolving treatment setting. Multiple primary malignancies, whether synchronous or metachronous, represent one of these complex scenarios. Herein, we report a case of resectable adenocarcinoma with a synchronous platelet-derived growth factor receptor A (*PDGFRA)*-rearranged myeloid neoplasm treated with a combination of neoadjuvant chemoimmunotherapy and imatinib, resulting in a complete pathologic response at the time of surgery.

## Case presentation

**Figure 1:**
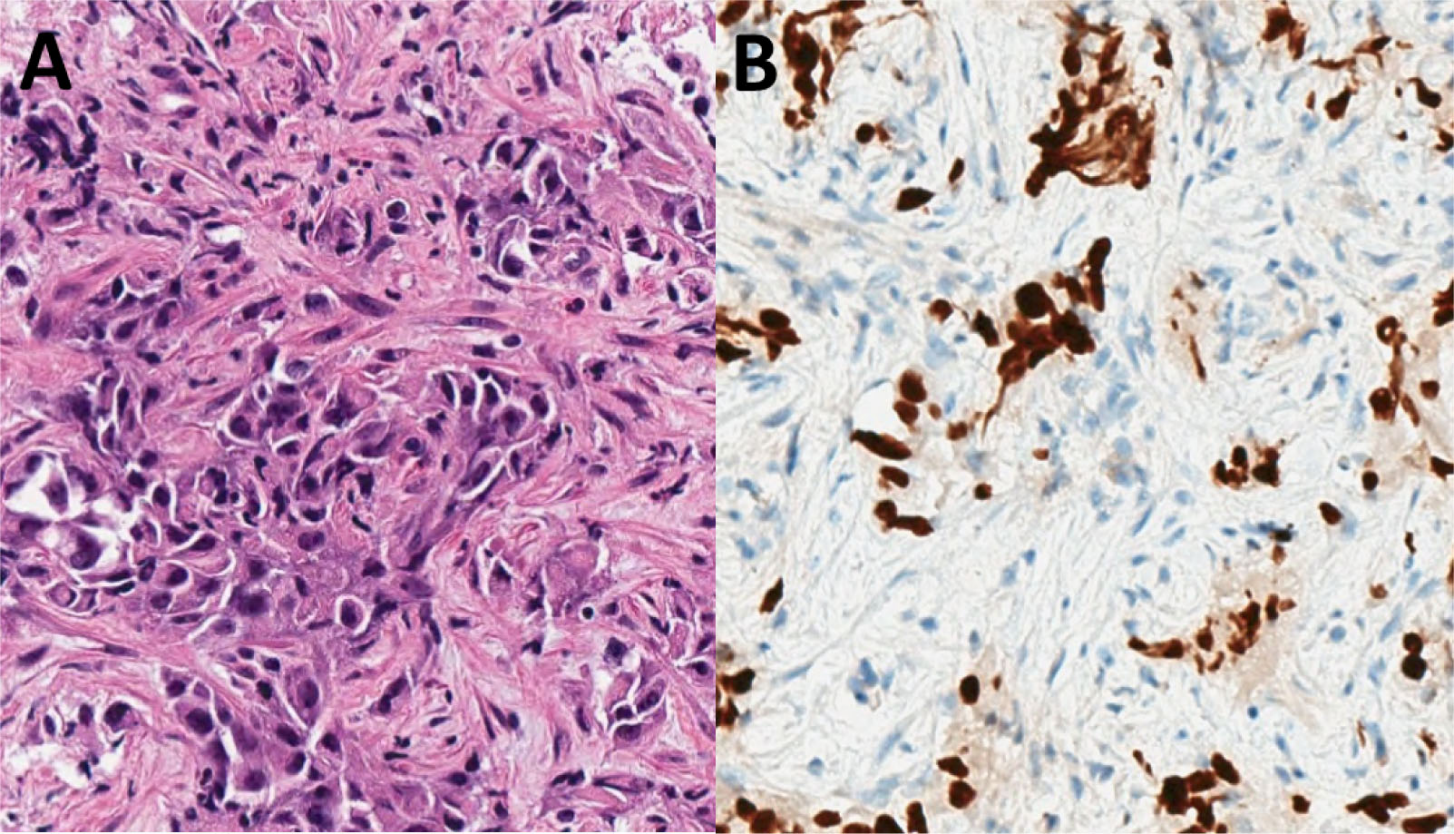
Initial biopsy pathology: **(A)** Transbronchial needle aspirate of a station 7 lymph node demonstrated metastatic carcinoma (hematoxylin and eosin, high power). **(B)** An immunohistochemical stain for TTF-1 was positive in the tumor cells, consistent with a diagnosis of adenocarcinoma (TTF-1 clone SP141, high power).

A 57-year-old man, active smoker, with a 25 pack-year smoking history, chronic obstructive pulmonary disease, and asthma, initially presented with productive cough and shortness of breath. Initial chest computed tomography revealed new lingular consolidation with mediastinal and bilateral hilar lymphadenopathy. Follow-up positron emission–computed tomography (PET-CT) scan revealed a hypermetabolic 2.4-cm lingular lesion, hypermetabolic subcarinal and aortopulmonary lymph nodes, and diffuse hypermetabolic bone marrow uptake involving the axial and appendicular skeleton. After several months of delay, the patient underwent endobronchial ultrasound (EBUS) with biopsy of lymph node stations 4R, 7, 11Ri, and 11Rs. Pathology demonstrated poorly differentiated adenocarcinoma involving lymph node station 7, with the remaining sampled lymph nodes negative for malignancy (**Figure 1**), consistent with stage IIIA disease (pT1cN2M0). Staging brain magnetic resonance imaging was negative for intracranial disease.

The quantity of tissue from these biopsy samples was insufficient to perform appropriate ancillary molecular testing; thus, programmed-death ligand 1 (PD-L1) status was unknown. Single-gene testing for *EGFR* and *ALK* alterations with polymerase chain reaction and fluorescence *in situ* hybridization (FISH) were requested; however, tissue was again insufficient to complete testing. A repeat EBUS to obtain additional tissue for molecular testing was discussed; however, the patient declined, wishing to avoid further procedures. Therefore, liquid next-generation sequencing of circulating tumor DNA (ctDNA) was performed, which did not reveal any targetable alterations, only a synonymous *PTEN* mutation.

During his preoperative work-up, the patient was found to have eosinophilia, with white blood cell (WBC) counts ranging from 20,000 to 25,000/mcL, and increased eosinophils (45%–55%). A *FIP1L1-PDGFRA* rearrangement was identified through peripheral blood FISH. Subsequent bone marrow biopsy was focally hypercellular (70%) with right-shifted granulopoiesis and predominance of eosinophils, with no dyspoiesis or increase in blasts. The karyotype was normal; however, in light of the peripheral blood FISH results, the findings were consistent with a myeloid neoplasm with *PDGFRA* rearrangement.

**Figure 2:**
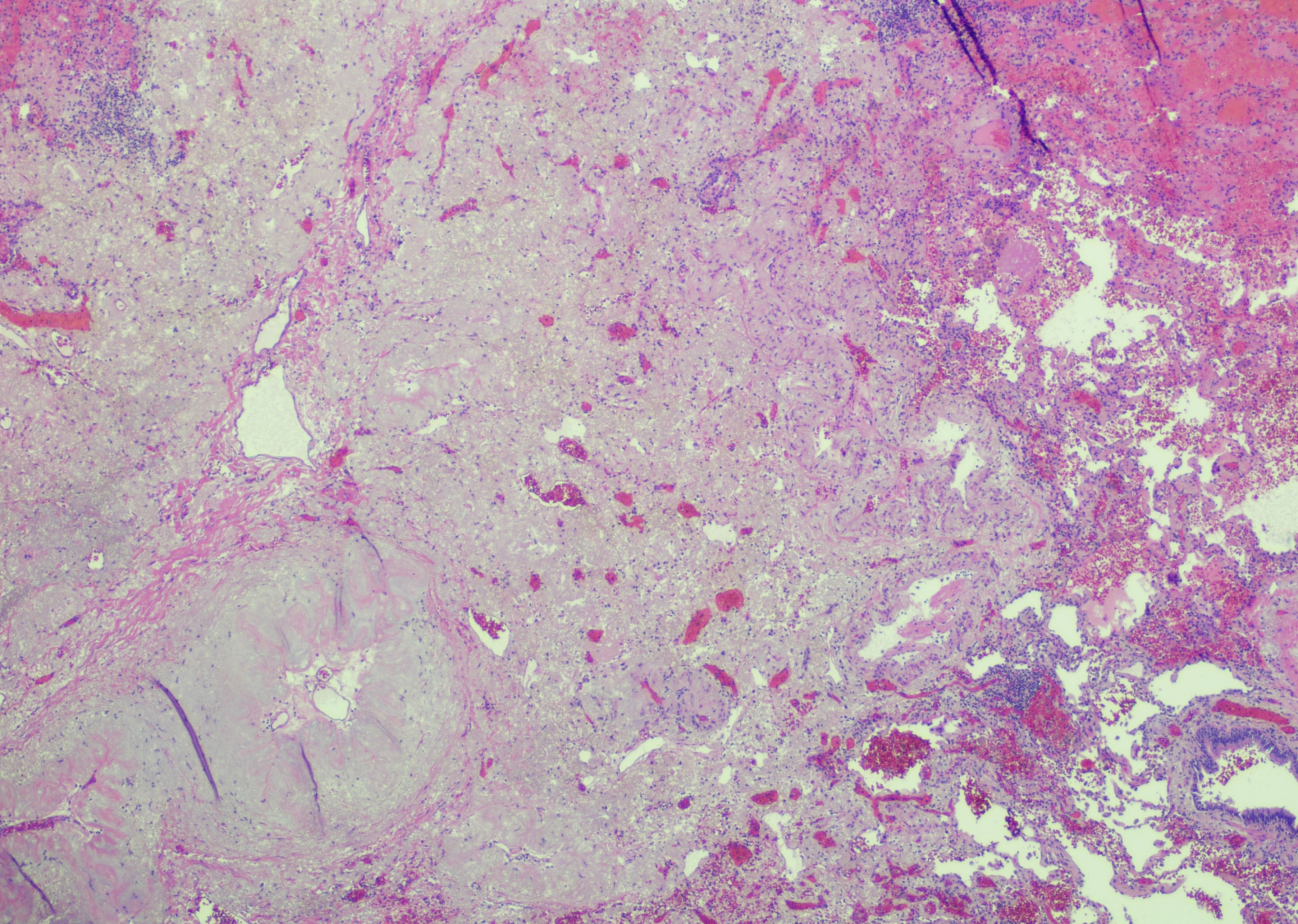
Resection pathology: Evaluation of the post-neoadjuvant therapy lobectomy specimen revealed a tumor bed with fibrosis, hemorrhage, and reactive epithelial cells; no residual viable tumor was present (hematoxylin and eosin, low power).

After extensive multidisciplinary discussion with malignant hematology, thoracic surgery, and thoracic medical oncology, the patient was started on neoadjuvant platinum-doublet chemotherapy and nivolumab, along with imatinib 100 mg for treatment of his eosinophilia. The patient completed his three preoperative cycles of chemoimmunotherapy without significant toxicity. An interval PET-CT scan showed a decrease in size of the lesion to 1.4 cm and no metabolic activity in either the primary lingular nodule or the previously identified hypermetabolic lymphadenopathy, along with resolution of the previously described hypermetabolic bone marrow uptake. Six weeks later—five months after initiating treatment—the patient was taken to the operating room for a left upper lobe lobectomy with lymph node dissection at levels 5L, 7, 8L, 9L, 10L, 11L, and 12L. Pathologic review of the surgical resection specimen revealed a pathologic complete response (pCR) (**Figure 2**).

**Figure 3:**
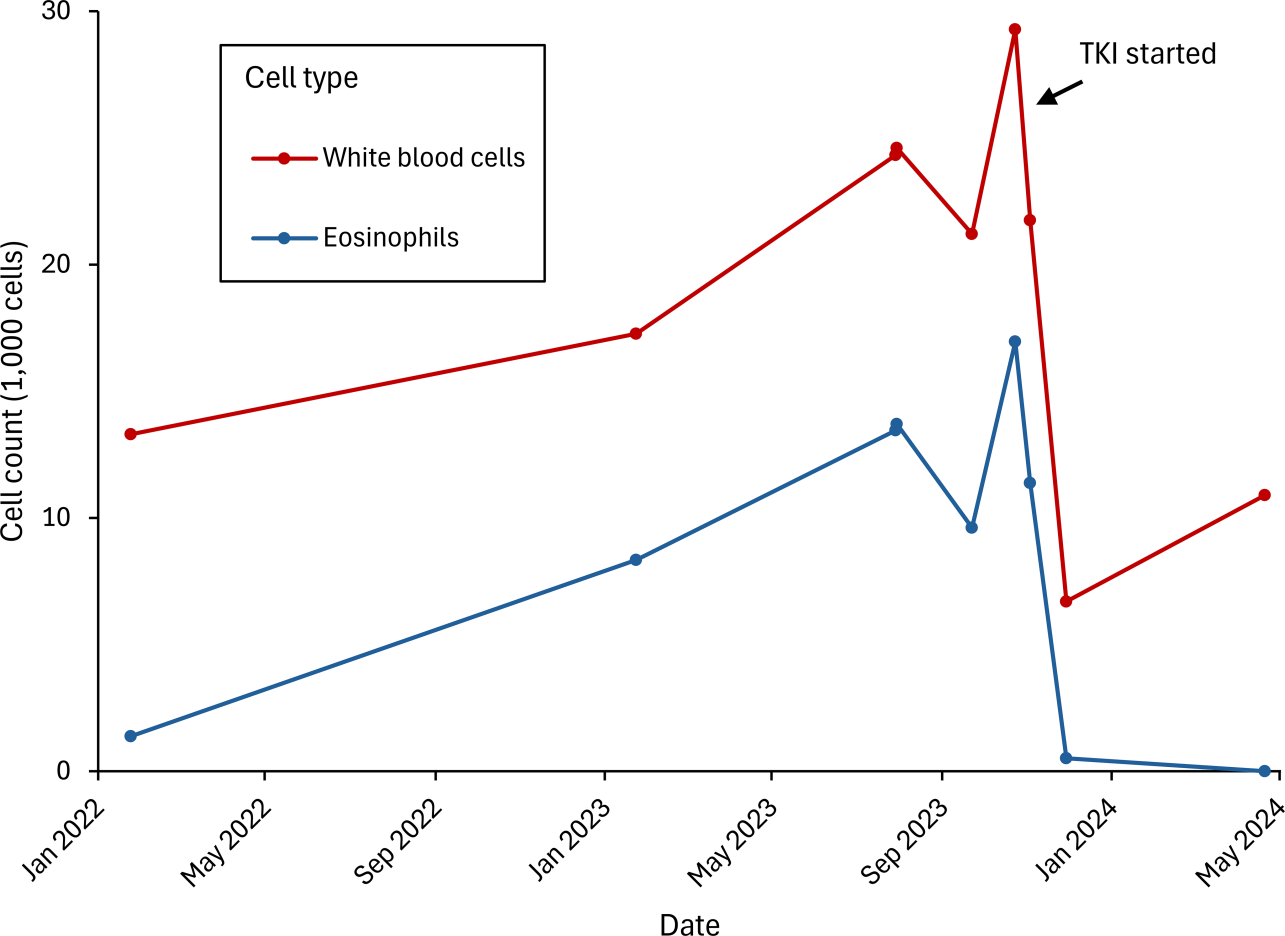
White blood cell and eosinophil counts over time.

His eosinophilia rapidly resolved following initiation of imatinib. The peak absolute eosinophil count was 16,500/mcL, which decreased to 520/mcL after two weeks of treatment and to 0 after four months. The patient continues his postoperative management with surveillance imaging every three months, per National Comprehensive Cancer Network guidelines, without evidence of recurrence on CT at 12-month follow-up. For his myeloid neoplasm, he remains on imatinib 100 mg, with no eosinophilia or leukocytosis (**Figure 3**).

## Discussion and conclusions

This report represents a real-world scenario involving the management of a patient with newly diagnosed resectable lung adenocarcinoma with co-occurring eosinophilia from a myeloid neoplasm with *PDGFRA* rearrangement. An additional complicating factor was limited tissue availability, which impacted biomarker testing. Despite these challenges, the patient initiated neoadjuvant chemoimmunotherapy with concurrent low-dose imatinib and ultimately underwent an uncomplicated and successful definitive resection with pCR. The management of patients with resectable NSCLC outside the clinical trial setting, particularly those with competing comorbidities such as multiple active malignancies, remains an ongoing challenge where continued real-world data and clinical experience will help provide guidance.

In our report, the decision to proceed with neoadjuvant therapy despite the simultaneous myeloid neoplasm was made only after significant multidisciplinary discussion and consideration of the prognoses associated with the patient’s myeloid neoplasm and resectable NSCLC. Although rare, myeloid neoplasms with *PDGFRA* rearrangement have an excellent prognosis, and complete, durable remission can be achieved with low-dose imatinib (5, 6). Depending on the nature of the specific simultaneous malignancy, such aggressive neoadjuvant strategies may not be appropriate. Therefore, case-by-case discussions are warranted.

Additionally, the use of imatinib in combination with chemoimmunotherapy was carefully considered. Previous data evaluated anti-programmed cell death protein 1 (anti-PD-1) therapy in combination with imatinib and other *BCR-ABL* tyrosine kinase inhibitors (TKIs) provided reassuring safety findings, which allowed us to proceed with this concurrent treatment strategy (7). There is some evidence that anti-PD-1 therapy in combination with dasatinib leads to upregulation of MHC class II and a potential synergistic anticancer effect (8). This contrasts with other TKI therapies, where combinations with ICB may increase the risk of immune-mediated toxicity (9); therefore, careful deliberation—ideally supported by preliminary safety data—should be exercised before initiating such combinations outside a clinical trial setting.

In biomarker testing, limited tissue availability is a common issue encountered in the metastatic disease setting, and with the increasing approval of perioperative therapies, it will become more frequent in early-stage settings as well. Factors such as patient willingness to undergo repeat biopsy, the risk associated with repeated procedures, urgency of treatment initiation, and smoking status all need to be weighed. For our patient, in light of his significant smoking history, expressed desire to avoid repeat bronchoscopy, prolonged delays in diagnostic work-up, and negative ctDNA results, we collectively decided to proceed with neoadjuvant chemoimmunotherapy. However, for a patient more willing for repeat biopsy or one with limited or no smoking history, the decision may have been different. Navigating such scenarios will become increasingly important for clinicians as these therapies are adopted into standard-of-care practice.

In conclusion, we present a case of co-occurring lung adenocarcinoma and eosinophilic myeloid neoplasm treated with a combination of neoadjuvant chemoimmunotherapy and low-dose imatinib, resulting in an uncomplicated clinical course, complete pathologic response after resection, and normalization of cell counts. This case serves as a successful example of the use of neoadjuvant therapy despite competing comorbid conditions. Clinical strategies will need to be developed and shared as neoadjuvant treatment approaches become more commonly used in real-world settings.

## Data Availability

The original contributions presented in the study are included in the article/Supplementary Material. Further inquiries can be directed to the corresponding author.
